# Adoptive T Cell Therapy for Epstein–Barr Virus Complications in Patients With Primary Immunodeficiency Disorders

**DOI:** 10.3389/fimmu.2018.00556

**Published:** 2018-03-19

**Authors:** Lauren P. McLaughlin, Catherine M. Bollard, Michael D. Keller

**Affiliations:** ^1^Center for Cancer and Immunology Research, Children’s National Health System, The George Washington University, Washington, DC, United States; ^2^Division of Oncology, Children’s National Health System, Washington, DC, United States; ^3^Division of Allergy and Immunology, Children’s National Health System, Washington, DC, United States; ^4^Division of Blood and Marrow Transplantation, Children’s National Health System, Washington, DC, United States

**Keywords:** primary immunodeficiency disorders, Epstein–barr virus, adoptive T cell therapy, immunotherapy, hematopoietic stem cell transplantation

## Abstract

Patients with primary immunodeficiency disorders (PID) have an increased risk from acute and chronic Epstein–Barr Virus (EBV) viral infections and EBV-associated malignancies. Hematopoietic stem cell transplantation (HSCT) is a curative strategy for many patients with PID, but EBV-related complications are common in the immediate post-transplant period due to delayed reconstitution of T cell immunity. Adoptive T cell therapy with EBV-specific T cells is a promising therapeutic strategy for patients with PID both before and after HSCT. Here we review the methods used to manufacture EBV-specific T cells, the clinical outcomes, and the ongoing challenges for future development of the strategy.

## Background

Epstein–Barr Virus (EBV) is a herpes virus that typically causes a mild to moderate self-limiting viral illness in healthy individuals. During primary infection, EBV establishes latency in B lymphocytes and oral epithelial cells. The level of B lymphocytes latently infected is maintained at a very low level through a potent cell-mediated immune response by EBV-specific T lymphocytes (1). However, individuals with moderate to severe forms of primary immunodeficiency disorders (PID) have weakened T-cell immunity with diminished immunosurveillance. PID patients are at risk from EBV-related complications which include acute and chronic infections and EBV-associated malignancies. EBV is also a frequent inciting factor for hemophagocytic lymphohistiocytosis (HLH) in PID with impaired cell-mediated cytotoxicity.

Hematopoietic stem cell transplantation (HSCT) has been used as curative approach for severe combined immunodeficiency (SCID) for over 50 years, and the approach is increasingly being used for other PIDs (2, 3). However, reconstitution of T cell immunity, needed to control both acquired viral infections reactivating viruses, is delayed for up to 6 months after transplantation. During this period patients remain extremely vulnerable to viral complications. While antiviral pharmacotherapy is available for many of the viruses that contribute to pre- and post-HSCT morbidity and mortality, their use is limited by toxicities and emerging resistance. Rituximab, a monoclonal antibody targeting CD20, has good efficacy against EBV. However, Rituximab targets not only the EBV-infected B cells, but also the healthy B cell compartments, which further weakens the immune system. Resistance to rituximab has also been described (4). Given these limitations, adoptive therapy with EBV-specific T cells has emerged as a promising therapeutic strategy for PID patients with EBV-related complications.

Adoptive therapy with viral-specific T cells (VSTs) has been used for over 20 years (5, 6). Earliest experience using cellular therapy for EBV-related post-transplant lymphoproliferative disease (PTLD) after HSCT included using unmanipulated donor lymphocyte infusions, which was often effective, but carried a high risk of graft-versus-host disease (GVHD) (7). Subsequently, VSTs have been developed that show safety and efficacy in treating EBV infections while minimizing the risk of GVHD (8–13). While previous reviews have primarily examined the use of all forms of VSTs for patients with PID (14, 15), this review focuses specifically on the development of and clinical use of EBV-specific T cells for patients with PID.

## EBV-Specific T Cell Generation Methods

Several methods have been developed to generate EBV-specific T cell products with minimally alloreactive T cells to decrease the risk of GVHD. These techniques include *ex vivo* expansion, multimer selection, and IFN-γ capture. To date, *ex vivo* expansion is the most commonly used method.

Many *ex vivo* expansion methods use EBV-transformed lymphoblastoid cell lines (LCL) as antigen presenting cells (APCs). LCLs are advantageous APCs as they express all 10 EBV latency antigens (type III latency), but also high levels of class I and II HLA and co-stimulatory molecules (16). Either activated monocytes or dendritic cells are used in the first stimulation, with LCLs used for subsequent stimulations. To further refine this technique, groups have developed methods for modifying LCL by either pulsing with synthetic peptide pools encompassing viral antigens, or transducing LCLs with adenovirus vectors that overexpress either latent membrane protein (LMP) 2 or LMP1 and LMP2. These strategies enhance T cell specificities for the less immunogenic EBV antigens LMP1 and LMP2 increasing their efficacy for EBV-related lymphomas that only express LMP1 and LMP2 (type II latency). While this method has proven to be safe and efficacious, it takes at least 8 weeks to generate a product suitable for clinical use as LCL take 3–4 weeks to manufacture. This has spurred the development of rapid *ex vivo* culture methods using a single stimulation with APC pulsed with synthetic peptide pools, or direct stimulation of PBMCs with synthetic peptide pools. These methods reduce the manufacturing time to 10–14 days. Rapid *ex vivo* culture methods have been used for multivirus specific T cells, but not for T cell products specific for EBV only.

Additional techniques, such as multimer selection or IFN-γ capture, can produce VSTs even more readily than rapid *ex vivo* culture (17–19). Multimer selection uses magnetically labeled peptide multimers to isolate T cells specific for the relevant peptide/MHC multimers. IFN-γ capture uses an immunomagnetic separation device to isolate T cells that produce IFN-γ when stimulated by viral antigens. Although these techniques produce a clinical grade product within 48 h, they require donors not only to be seropositive to the virus of interest, but also to have a detectable level of circulating virus specific T cells. Leukapheresis is typically needed to collect enough T cells for clinical use. While IFN-γ capture is not HLA-restricted and produces a polyclonal and polyfunctional product containing CD4^+^ and CD8^+^ T cells, multimer selection is an HLA-restricted process, and generally yields only CD8^+^ T cells.

## Previous Clinical Usage of EBV-Specific T Cells for PID Disorders

### Donor-Derived EBV-Specific T Cells

As PID is one of the most common non-malignant indications for referral to HSCT in pediatrics and is associated with high risk for viral complications, patients with PID constitute a sizeable proportion of patients in VST clinical trials (Table 1). A large, multi-center study with a median follow-up of 10 years treated 114 patients with EBV-specific T cells after HSCT, either for prophylaxis (*n* = 101) or treatment (*n* = 13) and included 13 patients with PID. All patients treated as prophylaxis had no subsequent EBV viremia, while three patients with active disease attained a complete response (CR) and three additional patients achieved a PR (10). Papadopoulou et al. included four patients with PID in their clinical trial of multivirus-specific T cells (CMV, EBV, AdV, HHV6, BK), two of whom received T cells for EBV-related complications and both of whom obtained a CR (20).

**Table 1:** Previous clinical use of donor-derived EBV-specific T cells.

Reference	Primary immunodeficiency disorders diagnosis	Indication	Specificity	Generation method	Source	Cell Dose	Outcomes
Leen et al. (21)	SCID	Prophylaxis	EBV, AdV	Culture, lymphoblastoid cell lines (LCL) with Ad5f35 vector	Hematopoietic stem cell transplantation (HSCT) donor, peripheral blood	1.35 × 10^8^/m^2^	Alive, no active infections
Papadopoulos et al. (7)	GATA2 deficiency	EBV, BK	CMV, EBV, AdV, HHV6, BK	Culture, peptide	HSCT donor, peripheral blood	2 × 10^7^/m^2^	CR
	SCID variant	BK, EBV	CMV, EBV, AdV, HHV6, BK	Culture, peptide	HSCT donor, peripheral blood	2 × 10^7^/m^2^	CR
	HLH	HHV6, BK; subsequent EBV reactivation	CMV, EBV, AdV, HHV6, BK	Culture, peptide	HSCT donor, peripheral blood	1 × 10^7^/m^2^	HHV6: CR; BK: NR; EBV: CR
Heslop et al. (10)	XLP	Prophylaxis	EBV	Culture, LCL	HSCT donor, peripheral blood	2 × 10^7^/m^2^	No viremia
	CID	Prophylaxis	EBV	Culture, LCL	HSCT donor, peripheral blood	2.5 × 10^7^/m^2^	No viremia
	WAS	Prophylaxis	EBV	Culture, LCL	HSCT donor, peripheral blood	2.5 × 10^7^/m^2^	No viremia
	XLP	Prophylaxis	EBV	Culture, LCL	HSCT donor, peripheral blood	2 × 10^7^/m^2^	No viremia
	XLP-like	Prophylaxis	EBV	Culture, LCL	HSCT donor, peripheral blood	2 × 10^7^/m^2^	No viremia
	WAS	EBV viremia	EBV	Culture, LCL	HSCT donor, peripheral blood	2 × 10^7^/m^2^	CR
	SCAEBV/NK defect	EBV viremia	EBV	Culture, LCL	HSCT donor, peripheral blood	1 × 10^8^/m^2^	CR
	SCAEBV	EBV viremia	EBV	Culture, LCL	HSCT donor, peripheral blood	2 × 10^7^/m^2^	PR; died of progressive lymphoma
	SCAEBV	EBV viremia	EBV	Culture, LCL	HSCT donor, peripheral blood	2 × 10^7^/m^2^	No further EBV reactivation
	XLP (SLAM mutation)	EBV viremia	EBV	Culture, LCL	HSCT donor, peripheral blood	2 × 10^7^/m^2^	CR
	XLP	EBV viremia	EBV	Culture, LCL	HSCT donor, peripheral blood	2 × 10^7^/m^2^	PR
	XLP	EBV viremia	EBV	Culture, LCL	HSCT donor, peripheral blood	2 × 10^7^/m^2^	CR
	XLP	EBV viremia	EBV	Culture, LCL	HSCT donor, peripheral blood	2 × 10^7^/m^2^	PR
Doubrovina et al. (12)	XLP	EBV-LPD	EBV	Culture, LCL	HSCT donor, peripheral blood	1 × 10^6^/kg × 3 doses	PD; died
	ALPS	EBV-LPD	EBV	Culture, LCL	HSCT donor, peripheral blood	1 × 10^6^/kg	NE; died
Naik et al. (14)	IL2RG-SCID	Prophylaxis	CMV, EBV, AdV	Culture, DC, and LCL with Ad5f35f-CMVpp65 vector	HSCT donor, umbilical cord	1.5 × 10^7^/m^2^	No viremia
	IL2RG-SCID	Prophylaxis	CMV, EBV, AdV	Culture, DC, and LCL with Ad5f35f-CMVpp65 vector	HSCT donor, umbilical cord	2.5 × 10^7^/m^2^	No viremia
	IL2RG-SCID	Prophylaxis	CMV, EBV, AdV	Culture, DC, and LCL with Ad5f35f-CMVpp65 vector	HSCT donor, umbilical cord	1 × 10^7^/m^2^	No viremia
	IL2RG-SCID	Prophylaxis	CMV, EBV, AdV	Culture, DC, and LCL with Ad5f35f-CMVpp65 vector	HSCT donor, umbilical cord	1 × 10^7^/m^2^	No viremia
	WAS	Prophylaxis	CMV, EBV, AdV	Culture, DC, and LCL with Ad5f35f-CMVpp65 vector	HSCT donor	1 × 10^7^/m^2^	No viremia
CID	Prophylaxis	EBV	Culture, peptide	HSCT donor	2.5 × 10^7^/m^2^	No viremia
HLH (STXBP2)	CMV, EBV	CMV, EBV, AdV	Culture	HSCT donor	1 × 10^7^/m^2^ × 2 doses	CMV: CR; EBV: CR
WAS	Prophylaxis	CMV, EBV, AdV	Culture	HSCT donor	2 × 10^7^/m^2^	No viremia

*SCID, severe combine immunodeficiency; EBV, Epstein–barr virus; AdV, adenovirus; CMV, cytomegalovirus; HHV6, human herpesvirus 6; CR, complete response, HLH, hemophagocytic lymphohistiocytosis; NR, no response; XLP, X-linked lymphoproliferative disease; CID, combined immune deficiency; WAS, Wiskott–Aldrich syndrome; SCAEBV, severe chronic active EBV; ALPS, autoimmune lymphoproliferative syndrome; DC, dendritic cell*.

In a large retrospective review of 36 PID patients receiving VSTs, Naik et al. included four patients with IL2RG-SCID as well as patients with Wiscott–Aldrich and combined immunodeficiency disorder (CID) who received donor-derived-specific T cells for prophylaxis. All patients remained free of EBV viremia after receiving T cells (14). Additionally, one patient with HLH received donor-derived trivirus VSTs (CMV, EBV, Adv) for CMV and EBV viremia with clearance of both viruses.

### Third Party EBV-Specific T Cells

To make cellular therapy more readily available, there is growing interest in establishing third-party banks of VSTs. Such T cell therapeutics produced from healthy donors are available for “off-the-shelf” use, eliminating the time and cost associated with custom-made products. These would be particularly beneficial in the setting of T-cell depleted transplantation, or when EBV-naive donors are the sole option for an EBV-seropositive patient, which would impart high risk of viral reactivation particularly in those with prior EBV-associated disease. While there is limited experience with third party banks to date, the results have been promising, particularly in patients with PID (Table 2).

**Table 2:** Previous clinical use of third party Epstein–Barr Virus (EBV)-specific T cells.

Reference	Primary immunodeficiency disorders diagnosis	Indication	Specificity	Generation method	Source	Cell dose	Outcomes
Vickers et al. (22)	Combined immunodeficiency disorder (CID)	PTLD	EBV	Culture, LCL	Third party	1–2 × 10^6^/kg/dose; 4 doses given weekly	CR
	CGD	PTLD	EBV	Culture, LCL	Third party	1–2 × 10^6^/kg/dose; 4 doses given weekly	PD; died
	CID	PTLD	EBV	Culture, LCL	Third party	1–2 × 10^6^/kg/dose; 4 doses given weekly	PD; died
Wynn et al. (23)	CTPS1 deficiency	Primary CNS lymphoma	EBV	Culture, LCL	Third party	2 × 10^6^/kg/dose; 7 doses given weekly; 2 additional doses after re-emergence of EBV disease	CR
Doubrovina et al. (12)	Hemophagocytic lymphohistiocytosis (HLH)	EBV-LPD	EBV	Culture, LCL	Third party	1 × 10^6^/kg x 3 doses	CR
Naik et al. (14)	ADA-severe combined immunodeficiency	EBV-LPD	CMV, EBV, AdV	Culture	Third party, pre-hematopoietic stem cell transplantation	5 × 10^6^/m^2^	NR, died from EBV-LPD
	HLH	EBV	EBV	Culture	Third party	2 × 10^6^/kg x 3 doses	PR; died of PTLD
	CTPS1 deficiency	EBV-LPD	EBV	Culture	Third party	2 × 10^6^/kg x 2 doses	CR
Withers et al. (24)	SCAEBV	EBV	EBV	Culture	Third party	2 × 10^7^/m^2^	EBV: NR; Died

*CGD, chronic granulomatous disease; PTLD, post-transplant lymphoproliferative disease; PD, progressive disease; CTPS1, CTP synthase 1 deficiency; EBV-LPD, EBV-lymphoproliferative disease; PR, partial response*.

Vickers et al. established a large third party bank of EBV-specific T cells to treat patients with PTLD and other EBV complications after HSCT or solid organ transplantation. To date, they have treated three patients with PIDs, including combined immune deficiency and chronic granulomatous disease (CGD). One patient had a CR, but the other two died from progressive disease (PD). At the time of publication, one additional patient with CGD had not undergone HSCT, but had EBV-specific T cells matched for use after transplantation (22). Two patients with CTP synthase 1 (CTPS1) deficiency have been treated with third party EBV-specific T cells for EBV-LPD and primary CNS lymphoma, and both had CRs after T cell therapy (14, 23). Third party EBV-specific T cells have also been used in HLH as discussed below.

## Special Cases

### Pretransplantation

As patients with PID are particularly vulnerable to chronic and refractory viral infections even prior to HSCT (25–27), another benefit of third party T cells is the ability to treat patients prior to transplant. This would not only minimize mortality associated with transplant, but could allow more patients to be referred to transplant. Naik et al. described two patients with PID (one with SCID and another with CTPS1 deficiency) with partially HLA-matched third-party T cells prior to HSCT for EBV-LPD, one of whom achieved a CR (14, 22, 23). A patient with ADA-SCID who developed EBV viremia and CMV colitis while on enzyme replacement therapy was treated with trivirus VSTs (CMV, EBV, AdV) that were 5/10 HLA matched with the patient, but no response was seen and the patient died of EBV-associated lymphoma. It was unclear if VST expansion may have been compromised in this case by the underlying inherent lymphotoxicity of ADA deficiency, as patients remain lymphopenic even in the setting of optimal enzyme replacement therapy. However, the patient with CTPS1 deficiency received 2 doses (2 × 10E^6^/kg/dose) of 9/10 HLA match EBV-specific T cells and attained a CR, and underwent subsequent HSCT without further viral complications.

### HLH With EBV Viremia

Hemophagocytic lymphohistiocytosis is condition of hyperinflammation associated with immune dysregulation secondary to defects in cytotoxic T lymphocyte and NK cell function. HLH can be either primary (associated with a known mutation) or secondary, and EBV viremia is a common trigger. In particular, familial HLH due to mutations in *STXBP2* and *PRF1* have been associated with chronic EBV viremia (28). Other PIDs have an increased risk of developing HLH, and several are associated with EBV viremia as well as including SAP deficiency, XIAP deficiency, ITK deficiency, and CD27 deficiency. While EBV typically infects B cells, EBV-related HLH is frequently associated with EBV-infected T cells, which presents therapeutic challenges (29). As familial HLH is universally fatal without HSCT as definitive treatment, these patients may benefit from EBV-directed cell therapy to restore EBV-specific immunity early after transplant.

A series of 49 patients who were treated for EBV-LPD following HSCT with donor lymphocyte infusion and/or EBV-specific VST included three patients with PID: one with autoimmune lymphoproliferative syndrome, one with x-linked lymphoproliferative disease, and another had primary HLH. There was an overall response of 68% following EBV-specific VST infusion, including a CR in the patient with HLH who received third party EBV-specific T cells (12). Similarly, Papadopoulou et al. reported a patient with HLH initially treated with multivirus VSTs for HHV6 and BK viremia who subsequently developed EBV reactivation and attained complete clearance of EBV viremia without other EBV-directed therapy after T cell infusion (20). The review by Naik et al. included two patients with HLH and EBV viremia, one of whom attained a CR while the other died of progressive EBV-PTLD (14).

### Severe Chronic Active EBV

Severe chronic active EBV (SCAEBV) infection is a lymphoproliferative disorder characterized by markedly high levels of EBV in blood and tissue that often presents with fever, lymphadenopathy, hepatic dysfunction, and thrombocytopenia (30). In SCAEBV, EBV can infect T and NK cells in addition to B cells. While the etiology of SCAEBV is often unknown, the underlying defect may represent a type of immunodeficiency. To date, HSCT has shown to be the most effective treatment for SCAEBV. As the primary problem in SCAEBV is an ineffective immune response to EBV, adoptive T cell therapy with EBV-specific T cells may be very advantageous in this patient population after HSCT. Heslop et al. included three patients with SCAEBV (one of whom had a known NK cell deficiency) in their trial of 114 patients receiving EBV-specific T cells. One patient was treated on the prophylactic arm and remained free of EBV viremia. Of the two patients with active disease at the time of T cell infusion, one patient attained a CR while the other died of progressive EBV-associated lymphoma (10). Withers et al. reported a patient with SCAEBV who received third party EBV-specific T cells but died Day + 14 after infusion of PD (24).

## Challenges and Future Directions

In spite of the successes with EBV-specific T-cells, there remain limitations in this approach for treatment of PID. Resistance to EBV-specific cellular therapy has been described in several prior studies, which may in some cases relate to viral escape mutations. In one study, a mutation in EBNA-3B enabled viral escape post-T-cell therapy (31). The targeted EBV antigens differ slightly between trials, and further studies of the lability of targeted epitopes will be crucial to improve the efficacy of EBV-specific T-cell therapy, particularly in the third-party setting. Best methods of partial HLA matching of third-party EBV-specific T-cells is also unclear, particularly in the setting of rare HLA alleles that are not known to mediate recognition of immunodominant EBV epitopes. EBV-specific T-cells are also subject to inactivation or killing by immunosuppressive therapies, such as corticosteroids, which limits their use in the setting of GVHD following HCT. Genetic modification of antigen-specific T-cells to render them resistant to glucocorticoids and calcineurin inhibitors may enable treatment of PTLD in spite of immunosuppressive therapy (32, 33).

It is also unclear if adoptive T-cell therapy will be effective prior to HCT in forms of PID in which APCs are impaired or absent. In a recent study, defects in the costimulatory receptor CD70 resulted in EBV-associated disease (34). CD70 defects could theoretically impair the ability of allogeneic T-cells to lyse infected target cells. EBV-specific T-cells have not been explored in patients with EBV-driven HLH prior to HCT, and is unclear whether partial restoration of cytotoxicity would be of any benefit in this hyper-inflammatory disorder.

Currently, manufacturing of EBV-specific T-cells requires a facility with the ability to meet regulatory guidelines for production of immune effector cells for clinical use. Selection methods allow make use of automated-closed systems, but are expensive and yield low cell numbers. *Ex vivo* expansion yields higher cell numbers, but requires expertise in more than minimal product manipulation. In both settings, an EBV-seropositive donor would need to be identified for product manufacturing. Third party T-cells circumvent these limitations, but similarly are limited by cost of bank generation and regulatory hurdles that limit widespread availability. New multicenter trials using regional banks will improve accessibility to studies using EBV-specific T-cells. With the recent FDA approval of two chimeric antigen receptor T-cell products, it is hoped that the use of antigen-specific T-cells may similarly be approved in the near future, enabling widespread accessibility to these products.

## Ethics Statement

This study was carried out in accordance with the recommendations of The National Institutes of Health. The referenced studies were approved by the local Institutional Review Boards. All subjects gave written informed consent in accordance with the Declaration of Helsinki.

## Author Contributions

LM prepared the first draft of this manuscript, while MK performed the literature review and compiled the data set of patients included in Tables 1 and 2. MK and CB both reviewed and edited the final manuscript.

## Conflict of Interest Statement

The authors declare that the research was conducted in the absence of any commercial or financial relationships that could be construed as a potential conflict of interest.
